# Accurate Estimation of Airborne Ultrasonic Time-of-Flight for Overlapping Echoes

**DOI:** 10.3390/s131115465

**Published:** 2013-11-12

**Authors:** Esther G. Sarabia, Jose R. Llata, Sandra Robla, Carlos Torre-Ferrero, Juan P. Oria

**Affiliations:** Electronic Technology and Automatic Systems Department, School of Industrial and Telecommunications Engineering, University of Cantabria, Avda. de Los Castros s/n 39005, Santander, Spain; E-Mails: llataj@unican.es (J.R.L.); sandra.robla@unican.es (S.R.); carlos.torre@unican.es (C.T.-F.); juan.perezoria@unican.es (J.P.O.)

**Keywords:** airborne ultrasounds, accurate time of flight, dynamic response model, hidden echo detection, hidden echo time of flight, overlapping echoes, robotics, autonomous navigation

## Abstract

In this work, an analysis of the transmission of ultrasonic signals generated by piezoelectric sensors for air applications is presented. Based on this analysis, an ultrasonic response model is obtained for its application to the recognition of objects and structured environments for navigation by autonomous mobile robots. This model enables the analysis of the ultrasonic response that is generated using a pair of sensors in transmitter-receiver configuration using the pulse-echo technique. This is very interesting for recognizing surfaces that simultaneously generate a multiple echo response. This model takes into account the effect of the radiation pattern, the resonant frequency of the sensor, the number of cycles of the excitation pulse, the dynamics of the sensor and the attenuation with distance in the medium. This model has been developed, programmed and verified through a battery of experimental tests. Using this model a new procedure for obtaining accurate time of flight is proposed. This new method is compared with traditional ones, such as threshold or correlation, to highlight its advantages and drawbacks. Finally the advantages of this method are demonstrated for calculating multiple times of flight when the echo is formed by several overlapping echoes.

## 1. Introduction

Ultrasonic sensors have been widely used for obstacle detection tasks in autonomous vehicle navigation. For these applications ultrasonic sensors have several advantages with respect other technologies, like vision cameras, time of flight cameras or infrared sensors, because they are low cost and lightweight and they have simple excitation stages. Besides they can be used in environmental conditions that are impossible for other sensor technologies such as, for example, in poorly illuminated environments or in presence of fumes.

Furthermore, it should be said that there are some disadvantages with the use of airborne ultrasonic sensors. For example, it is necessary to work at low frequency in order to reach working distances of about one meter. This is due to the attenuation of ultrasonic waves in air. The propagation of ultrasounds in air is affected by the properties of the medium and the frequency of the signal. Therefore, it is necessary to use low frequency sensors, which have low directionality. Moreover, we should add that ultrasounds display a degree of uncertainty due to the effect of the variation of environmental parameters on these signals. All these factors have contributed to the application of ultrasound in air being less developed than in other media such as liquids or solids.

In this work, the use of ultrasonic sensors in object identification and reconstruction tasks is enhanced, introducing procedures to minimize its drawbacks as much as possible.

The aim of this work is to help to solve the problems related to recognition of the environment for use in autonomous robot navigation based on information provided by ultrasonic sensors. This study is aimed at applications in mobile autonomous robot navigation in structured environments where it is necessary to recognize the elements of the environment and their position relative to the robot.

For the pulse-echo sensor excitation technique, the ultrasonic signal received from the simple structured environments, such as those formed by flat walls and corners, is modeled. The development of this model is proposed in order to facilitate the subsequent reverse task: the recognition of these situations during robot navigation.

## 2. Typical Ultrasonic Structure Used in Mobile Robotics

There are two main types of ultrasonic transducers to operate in air: piezoelectric and electrostatic.

Electrostatic transducers have high sensitivity and bandwidth but require higher voltage for polarization and operation. The principle of operation is by vibration of a charged membrane which changes the distance with respect to the backplate producing a change in the capacitance. If the load is constant the voltage will be proportional to the capacitance. Different studies have applied these transducers to estimate distances, generate environment maps, differentiate edges, corners and walls such as those by Kuc [1–3], Peremans [4] and Kleeman [5].

Piezoelectric transducers require low voltage excitation signals (10–20 Vrms), so the excitation stages are simpler and lower cost. The operating principle is based on the application of a voltage to a resonant ceramic glass, which makes it vibrate at a certain frequency. The bandwidth of a glass ceramic is limited to a few kHz. This situation limits the rise time of the pulse envelope to approximately 0.5 ms. Sensors can be found in a wide range of frequencies ranging from 20 kHz to a few hundreds of MHz. The advantage of this type of sensors is that they do not require bias voltage, allowing the use of simple control electronics. This advantage makes many manufacturers offer these transducers without excitation and capture stages, making it necessary to build these stages. These sensors are also widely used in construction of environment maps, localization and classification of objects by classifiers, such as in Benet [6], Martinez [7] and Llata [8].

Nowadays there is another technology based piezoelectric PVDF (polyvinylidene fluoride) forming a flexible membrane which can be given the desired shape for measurement transducers. These transducers have lower sensitivity than glass ceramic and are often used in applications at very short distances.

This work is intended for application in mobile robotics using a common sensor configuration formed by an emitter-receiver pair. The sensors selected for the experimental work were of piezoelectric type and they work at low frequency since one of the conditions is that the working range must be at least one meter.

**Figure 1.**
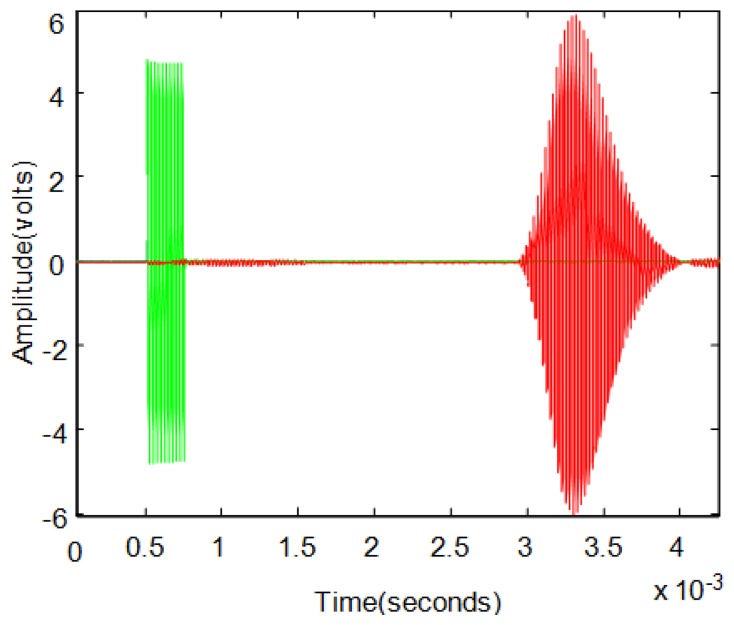
Example of excitation and echo signals.

The pulse-echo excitation technique is also commonly used in these applications. The emitter sensor excitation is formed by a pulse of several cycles of a sinusoidal signal. Applying this excitation signal to the transmitter causes the generation of an ultrasonic echo that travels through the air, bounces off the reflector surface and is captured by the sensor receiver. Figure 1 shows an example of the application of pulse-echo technique where you can see the excitation signal in green, and the captured echo signal in red.

The ultrasonic echo sensor captured by the receiver is formed by an amplitude-modulated signal which is a sinusoidal carrier at the resonant frequency of the sensor. In order to extract the information contained in the captured echo signals, preprocessing is required. It is very usual to use a Butterworth digital filter to cancel the carrier signal and to obtain the echo envelope. These filters have a nearly flat gain in the pass-band without ripple and are monotonically decreasing. The transfer function of a low-pass Butterworth analog filter is given by:

(1) $${\left|F(\omega )\right|}^{2}=\frac{1}{1+{\left(\frac{\omega }{\omega_{c}}\right)}^{2N}}$$

where *N* is the filter order, *ω* is the angular frequency and *ω_c_* is the cutoff frequency (−3 dB with respect to pass-band).

To convert the analog filter into a digital filter it is common to use the bilinear transformation with prewarping between the s-plane and the z-plane, guaranteeing the same response frequency for the selected frequency:

(2) $$s=n\frac{z-1}{z+1}$$

(3) $$n=\frac{\omega }{\text{tan}\frac{\omega T}{2}}$$

where *T* is the sampling period.

For example, for the case of a 40 kHz sensor the filter used could be designed as a third order low-pass Butterworth filter with a cutoff frequency of 5 kHz whose transfer functions would be:

(4) $$\frac{Y(z)}{X(z)}=\frac{\left(0.0376+0.1127z^{-1}+0.1127z^{-2}+0.0376z^{-3}\right)10^{-4}}{1-2.9372z^{-1}+2.8763z^{-2}-0.9391z^{-3}}$$

## 3. Emitter-to-Receiver Response Model

Several factors affect the emission and reception shape of the ultrasonic signal, for example the manufacturing technology, the method of integration of the components and the sensor encapsulation. Therefore to obtain a real signal model for the sensors, it is interesting to work with a more realistic model, instead of staying with the general approach of the response to the piston plane. This allows more complete analysis of the received echoes. So, for the case of driving the emitter via a pulsed type signal, a model of the transient response occurring during the transmission of the ultrasonic waves through the air is obtained. In this section, the characterization of the emitter-to-receiver temporal response of the ultrasonic sensors is performed, to be used in subsequent experimental development. This analysis will help us to obtain a better fit of the models of propagation and reflection.

### 3.1. Radiation Pattern Characterization

The propagation of the ultrasonic pressure *p* in the time *t* inside a fluid is given by the wave equation:

(5) $$\nabla^{2}p=\frac{1}{c^{2}}\frac{\partial^{2}p}{\partial t^{2}}$$

where *c* is the speed of sound.

Considering the spatiotemporal solution of the two-dimensional wave equation for a point source of spherical waves, and that the transmission of ultrasonic waves presents inversely proportional attenuation with the distance traveled, the pressure of an emitted wave *p_e_* can be represented by:

(6) $$p_{e}\left(t,\vec{r}\right)=\frac{P_{e}}{\left|\vec{r}\right|}e^{j\left(\omega t-k\vec{r}\right)}$$

where *k* is the wave number, *r* is the position vector defining the coordinates of the spatial point considered and *P_e_* is the amplitude of the emitted acoustic pressure.

As the transmission of ultrasonic waves is affected by the radiation pattern of the sensor, expression (6) can be completed taken into account this effect. According to [9], expression (6) corresponds to the pressure along the normal axis to the surface of the piston at a distance *r* from the sensor, and such that it fulfills the relation *r ≫ k*·a (where *a* is the radius of the piston) and where *P_e_*, depends on the density of the medium *ρ_0_*, the speed of sound *c* and the amplitude of the oscillation of the piston surface *U_0_* according to:

(7) $$P_{e}=\frac{1}{2}\rho_{0}cU_{0}ka^{2}$$

**Figure 2.**
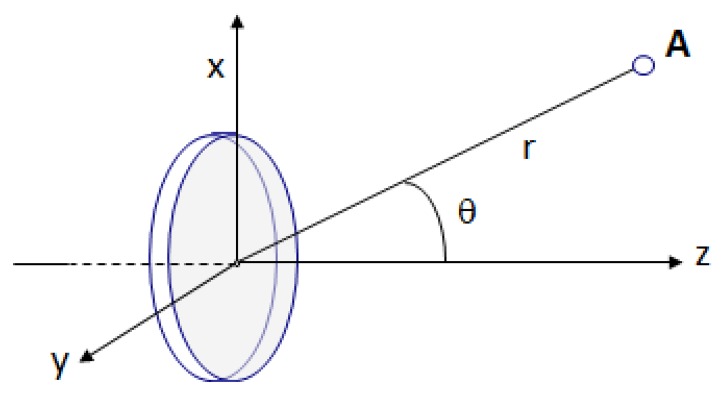
Point for analysis of the pressure outside the normal axis in polar coordinates.

To analyze the pressure *p* outside the normal axis to the piston at any point A, using polar coordinates *(r,θ)* as shown in Figure 2, we can add the directional factor *H(θ)*:

(8) $$H\left(\theta \right)=\frac{2J_{1}\left(\text{ka sin}\;\theta \right)}{\text{ka sin}\;\theta }$$

So, expression (6) can be completed for obtaining the pressure at any point:

(9) $$p\left(\vec{r},\theta ,t\right)=\frac{P_{e}}{\left|\vec{r}\right|}H\left(\theta \right)e^{j\left(\omega t-k\vec{r}\right)}$$

The real directional factor will vary with the frequency, the shape of the vibrating screen and the housing of the sensor. An experimental test was carried out to obtain the real radiation pattern of the transducers employed. For this test a Brüel Kjær omnidirectional free-field microphone type 4939 was used. These patterns give information about the shape of the radiation lobe and the attenuation which occurs due to the transmission medium in which the waves propagate.

To simplify the analysis, instead of using the directional factor *H(θ)* shown in expression (8), a Gaussian function is proposed to approximate the beam profile:

(10) $$A\left(\theta \right)=A_{0}e^{-\frac{\theta^{2}}{2\sigma^{2}}}$$

where A_0_ is the maximum amplitude in the axis sensor and σ is the beam width.

For example, using this expression, the directional factor for the SensComp 40LT16/40LR16 sensors is fitted experimentally as:

(11) $$H\left(\theta \right)=e^{-0.00085\theta^{2}}$$

So, for these sensors the complete expression will be:

(12) $$p\left(\vec{r},\theta ,t\right)=\frac{K}{\left|\vec{r}\right|}e^{-0.00085\theta^{2}}e^{j\left(\omega t-k\vec{r}\right)}$$

where *K* is a constant proportional to the peak amplitude of the excitation; the geometry and working frequency of the sensor; the density of the medium *ρ_0_*; and the speed of sound *c*.

**Figure 3.**
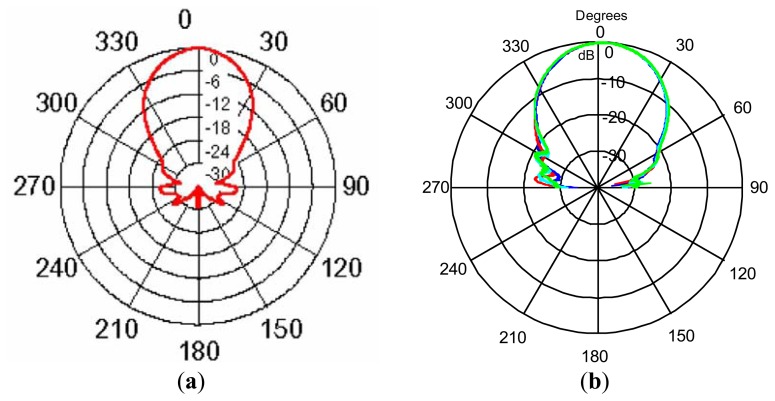
(**a**) Theoretical radiation pattern; (**b**) Real radiation pattern.

The experimental results for this sensor can be seen in Figure 3. Figure 3a shows the sound pressure received (in decibels) for angular scanning from −90 to +90 degrees, considering the axis of the sensor at zero degrees. Therefore, Figure 3a shows, in polar coordinates, the pattern given by the manufacturer and Figure 3b shows the real pattern obtained experimentally by a specially selected pattern microphone Brüel Kjær Type 4939, considering different measurement distances from the sensor housing surface (blue: 15 cm; red: 20 cm; cyan: 25 cm; green: 30 cm).

**Figure 4.**
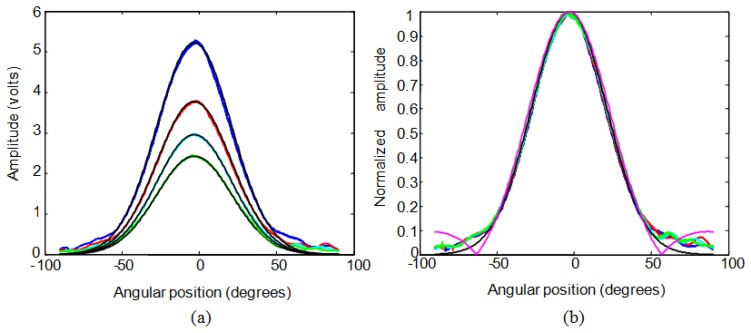
Angular position *versus* real and approximated pressure (**a**) without normalization (**b**) normalized.

**Figure 5.**
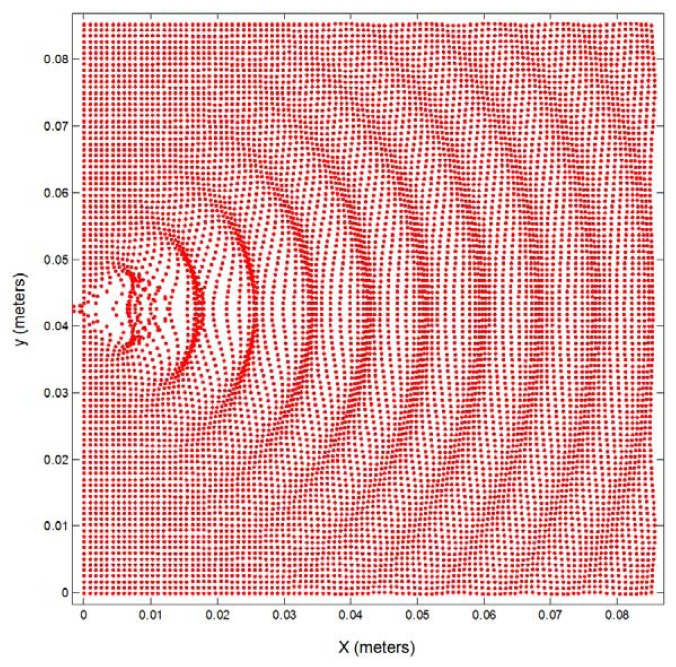
Point source emission at 40 kHz affected by the radiation pattern.

Figure 4a shows the experimental pressure data without normalization for different distances from this sensor (blue: 15 cm; red: 20 cm; cyan: 25 cm; green: 30 cm) for an angular sweep from −90 to +90 degrees. The same figure also shows, in a black line, the pressure calculated through the approximation 11. The figure demonstrates the similarity of the experimental data and the data approximated by expression (11). Figure 4b represents normalized experimental data together with the theoretical values, in magenta, calculated using expression (8), and the approximation, in black, according to expression (11). For the range from −50 to +50 degrees the three cases are very similar, so approximation 11 could be a good solution in order to avoid the complexity of expression (8). Figure 5 shows the two dimensional representation of the emission of an ultrasonic point source at 40 kHz using expression (12) from the origin to a distance of ten wavelengths.

### 3.2. Emitter-to-Receiver Time Transient Response

To perform the analysis described in the following sections, it is necessary to carry out a preliminary analysis of the temporal behavior of the transient response of the sensors to different driver inputs in order to obtain the range of excitation signals suitable for use in the pulse-echo technique.

So, in this section we analyze the transient response of these echoes generated by applying a sinusoidal excitation pulse to the transmitter sensor. The excitation signal pulses were formed by a succession of cycles of a sinusoidal signal at the resonant frequency of the sensor. A sweep from a minimum to a maximum number of excitation cycles was performed to examine its response.

Only the case of reflection on plane surfaces has been considered, because as stated by Nagy in [10] the effect of a little surface roughness is regarded as a weak perturbation of the known plane-wave solution involving the same refractive indexes and a smooth surface. This approximation is limited to slightly rough surfaces when the roughness parameter is small with respect to both the acoustic wavelength and the correlation length of the roughness. So, we assume this is the case of the walls that a mobile robot can find in its displacements in an indoor structured environment, where the walls and furniture can be smooth, textured or stippled. In this case the sensors employed operate at low frequency, 40 kHz, which corresponds to a wavelength of 8.5 mm, which is much larger than the dimensions of the possible roughness that those walls could have.

Instead of separately analyzing the dynamics of the transmitter and the receiver, and as they always work together in real applications, we have chosen to model the behavior of the transmitter-receiver pair as a single unit.

For the experimentation, the sensors are located at different distances and with different inclinations to take into account the effect of signal attenuation with distance and the effect to the radiation lobe of the sensor used. Under these conditions, several tests were carried out firstly with the transmitter facing the receiver, secondly by considering rebound on a normal flat wall, and finally rebound on an inclined flat wall. Different amplitude pulses were also considered to check the linearity of the sensor response. In the analysis performed, three different types of response can be seen.

**Figure 6.**
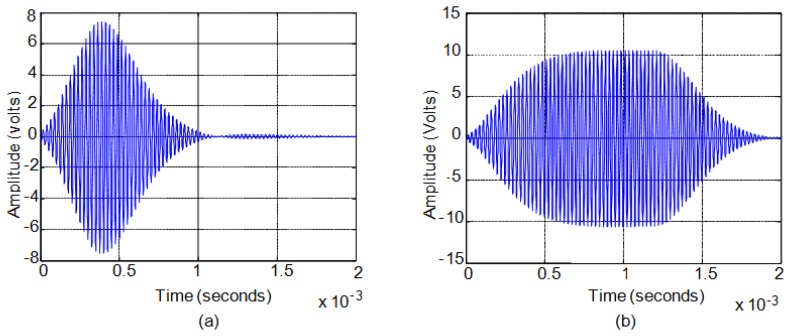
Echo generated for (**a**) short excitation pulse; (**b**) long excitation pulse.

First, when the excitation signals have duration of a few cycles the power of the transmitted signal is very low generating weak echoes that are difficult to analyze. Second, exceeding this threshold, when the duration of the excitation signal does not exceed the time it takes the sensor to reach steady-state response, the generated pressure always exhibits a maximum in its response. Figure 6a shows an example of this situation. Third, when the excitation pulse length is long, the sensor response reaches steady state and pressure remains stable until the excitation signal disappears. Figure 6b shows an example of this situation.

**Figure 7.**
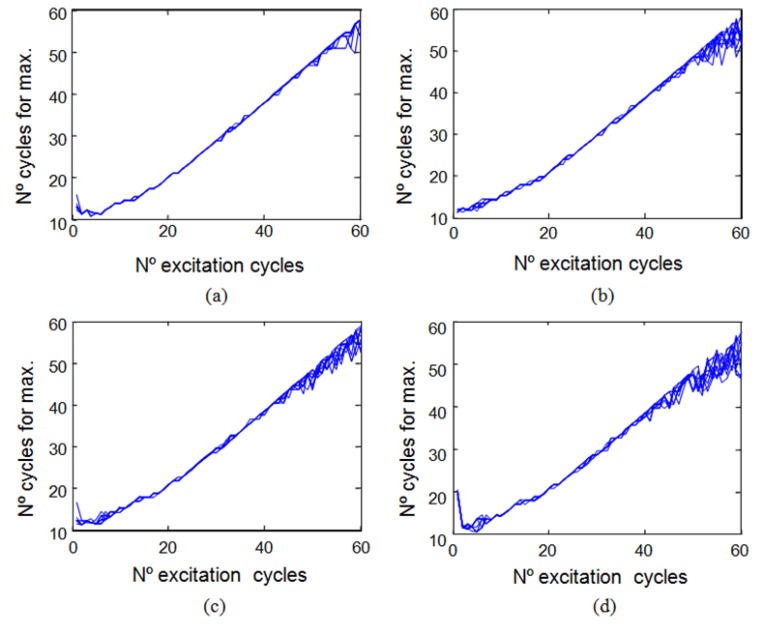
Excitation cycles *versus* cycles for maximum (**a**) transmitter and receiver facing; (**b**) Direct normal rebound; (**c**) Ten-degree-inclination wall rebound; (**d**) Twenty-degree inclination wall rebound.

In order to illustrate this analysis, for the Senscomp 40LT16/40LR16 sensors, the relationship between the number of cycles of the excitation signal and the cycle in which the maximum in the reception echo occurs can be seen in Figure 7. Figure 7a shows the case of transmitter and receiver facing each other with a separation of 40 cm between them. Figure 7b shows the case of direct rebound on a flat wall located at 40 cm and perpendicular to the axes of the sensors which are spaced three cm apart. Figure 7c shows the same case of rebound on a wall but with a ten-degree angle with respect to the emitter-receiver pair. Figure 7d shows the same case of rebound on a wall but with a twenty-degree angle with respect to the emitter-receiver pair.

In Figure 7 it can be seen that for very short excitation signals (less than six cycles for Senscomp 40LT16/40LR16 sensors) in the four cases shown there is no clear trend between the number of cycles of the excitation signal and the cycle in which the maximum occurs. This occurs because the echo signals are very weak. Therefore, this zone is not taken into consideration in the analysis.

When the pressure does not reach saturation, see Figure 6a, there is an almost linear relationship between the two variables analyzed, the number of cycles of the excitation signal and the cycle in which the maximum in the reception echo occurs. For the example shown in Figure 7, this range is comprised between six and fifty cycles of the excitation signal. In these cases, at the end of the excitation pulse the sensor has not reached the maximum pressure value that it can generate.

When the pressure reaches its stationary value before the excitation signal disappearing, see Figure 6b, the linear relationship is lost. In Figure 7, for over fifty cycles the ultrasonic wave cannot grow in amplitude and the response is stabilized to the maximum value reached becoming a stationary sinusoidal signal. The dispersed maximum values obtained for driving signal cycles over fifty cycles are due to disturbances when the output signal is already stationary, making the maximum occurs at a different point every time.

After analyzing the range of excitation signals suitable for the use with the pulse-echo technique, next we will obtain a model of the transient response of the echoes for this range of input signals.

First, we want to obtain a model of the response to short-duration excitation signals. This range is considered because it firstly enables the generation of transmitter pulses which do not reach sound pressure saturation, and secondly, it generates transient responses of short duration which help to avoid overlap with nearby echoes. Several simultaneously echoes often occur when the sensor lobe is very wide, as is the case of the low-frequency sensor used for experimentation. The sensor lobe covers a huge area and, therefore, the sensor is able to view multiple surfaces simultaneously.

The proposed model for short duration pulses corresponds to a second order system with a double real pole with a time constant *τ*. The expression for the envelope of the excitation signal for a pulse of *n* cycles of duration when 0 ≤ *t* ≤ *nT* is:

(13) $$x\left(t\right)=K_{1}$$

And the expression when *n* · *T* < *t* < ∞:

(14) x(t)=0

where *K_1_* is the amplitude of the excitation pulse.

The expression for the envelope of the response to a pulse of *n* cycles of duration when 0 ≤ *t* ≤ *nT* is is:

(15) $$y\left(t\right)=K_{1}K_{2}\;\left(1-\frac{1}{\tau }te^{\frac{-t}{\tau }}-e^{\frac{-t}{\tau }}\right)$$

And the expression when *n* · *T* < *t* < ∞:

(16) $$y\left(t\right)=K_{1}K_{2}\;\left(1-\frac{1}{\tau }te^{\frac{-t}{\tau }}-e^{\frac{-t}{\tau }}\right)-\left(1-\frac{1}{\tau }\left(t-nT\right)e^{-\frac{-\left(t-n\cdot T\right)}{\tau }}-e^{\frac{-\left(t-nT\right)}{\tau }}\right)$$

where *T* is the period of the wave and *K_2_* includes the gain due to the sensor itself and the gain due to the amplifiers used in the reception conditioning stage. For example, for Senscomp 40LT16/40LR16 sensors working with excitation signals from six to fourteen cycles, the time constant *τ* must be set to 160 μs.

**Figure 8.**
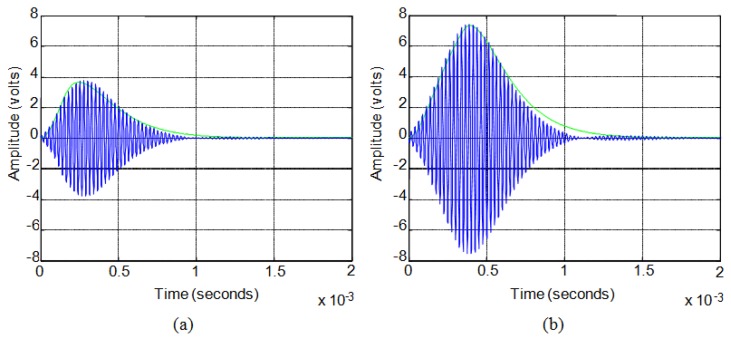
(**a**) Six-cycle pulse response; (**b**) Fourteen-cycle pulse response.

Figure 8 shows, for these sensors, the comparison between the actual experimental value of the response, in blue, and the approximate envelope obtained from the model, in green, for a sinusoidal excitation pulse of 5 V (10 Vpp). Figure 8a shows the case of a six-cycle pulse and Figure 8b the case of a fourteen-cycle pulse.

**Figure 9.**
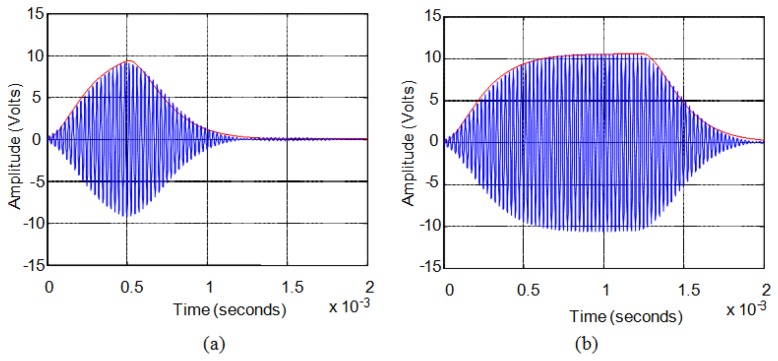
(**a**) Twenty-cycle pulse response; (**b**) Fifty-cycle pulse response.

In the case of long duration pulses the sensors have the same second order system behavior. For the same sensors, with excitation signals formed by more than fourteen cycles, this time constant *τ* must be set to 135 μs. Figure 9 shows the comparison between the actual experimental value of the response, in blue, and the approximate envelope obtained from the model, in red, for sinusoidal excitation pulse of 5 V (10 Vpp). Figure 9a shows the case of a twenty-cycle pulse and Figure 9b the case of a fifty-cycle pulse.

In summary, we can conclude that the behavior of the transient response of ultrasonic sensors is almost linear when the amplitude of the excitation signal is varied within the range limits provided by the manufacturer. Therefore, we have obtained a temporal response that, from the viewpoint of the echo envelope, is very like a second order overdamped system.

The sensors' behavior in relation to the number of cycles of the excitation signal is not linear but very close to be linear. We have proposed a linearized model that works correctly for short duration pulses, before reaching the sensors' sound pressure saturation limit, and another linearized model that works correctly for pulses where the transient response reaches the steady state when the sensor has reached sound pressure saturation. These models have been experimentally validated for a wide variety of real cases.

This approach and its mathematical implementation will allow us to better calculate the flight times, especially in complex situations when the full echo is formed by several overlapping sub-echoes due to simultaneous rebounds from various surfaces.

Thus, the propagation model represented by the expression (9) can now be completed with the dynamics of the transducer *y*(t), as shown below:

(17) $$P\left(\vec{r},\theta ,t\right)=\frac{K_{1}}{\left|\vec{r}\right|}\cdot y(t)\cdot H(\theta )\cdot e^{j\left(\omega t-k\vec{r}\right)}$$

## 4. Time of Flight Calculation Based on the Emitter to Receiver Transient Response

The time of flight is a simple measurement of the elapsed time between the start of the excitation of the transmitter sensor and the start of the echo received in the receiver sensor. This time can be easily translated into the distance that there is between sensors and objects in the surroundings. This distance is usually employed in autonomous robot navigation and also in environment mapping.

**Figure 10.**
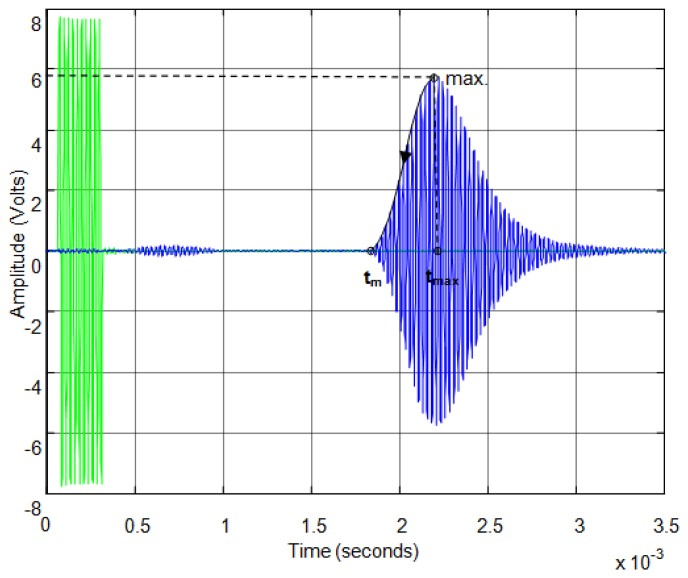
Example of emitted and received signal for a simple rebound.

Figure 10 shows an example of transmitted and received echo for a simple rebound. In this example we use a pair of sensors, one working as emitter and another working as receiver, three centimeters apart and facing a flat surface. The green signal is the excitation pulse formed by ten cycles of sinusoidal signal and the blue signal is the received echo after the rebound on the flat surface. In this figure it can observed that the first echo received is the noise due to the direct reception between emitter and receiver due to the proximity between them.

In this paper, we propose obtaining an accurate start time of the echo employing the transient response modeled in the previous section (see expressions (15) and (16)). Once we have approximated the response of the sensor to pulse excitation with a linear model, we can obtain the time constant of the second order model proposed and then we can estimate, knowing the time of the maximum of the echo t_max_, the start time of the echo t_m_ as Figure 10 shows.

### 4.1. Comparison of Time-Domain Methods for Calculating the Start Time of the Echo

Next, the proposed method for the calculation of the start time of the echo is compared to two classical time-domain methods. An updated summary of the techniques used for calculating the time of flight can be seen in [11,12]. For a single echo the start time is calculated by threshold, by correlation and by the proposed method. In order to define the threshold and the correlation as a percentage of the total value and to make a better comparison between the different methods, a normalized echo signals has been used in these cases. This implies that the full signal echo is necessary to implement any of the methods.

**Figure 11.**
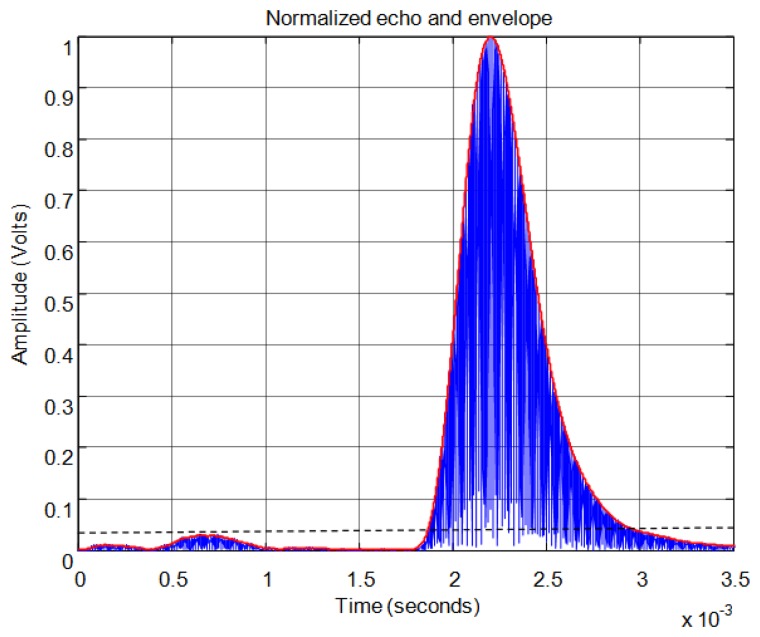
Normalized echo and envelope.

**Figure 12.**
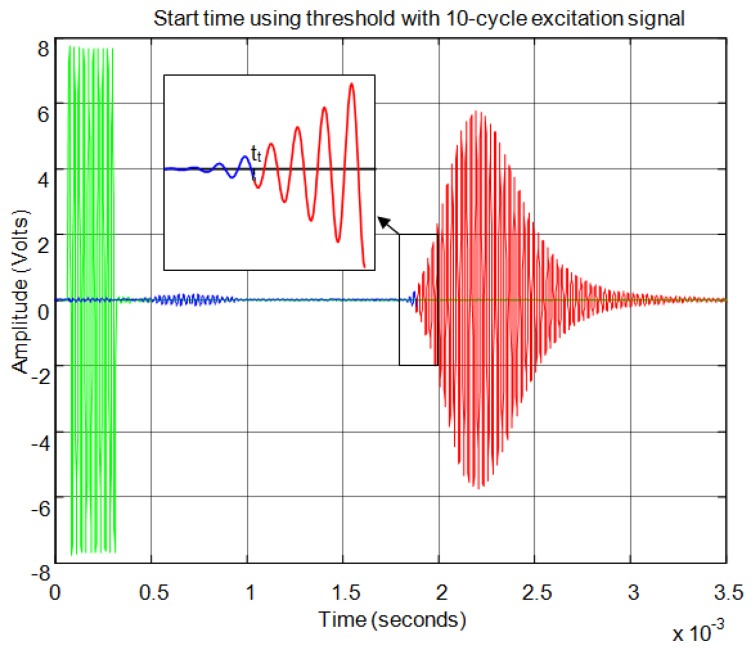
Time of flight detection using threshold.

For the threshold method, the signal must surpass the noise level in the received echo to set the minimum required signal in order to calculate the start time. In Figure 11 the echo (in blue) and the envelope (in red) of the received signal can be seen. In this figure the noise derived from the direct reception of the excitation signal located between 0.5 and 1 ms of the response can be observed. In this case, the threshold level must be at least 0.04 in the normalized response. With this threshold Figure 12 shows the point detected as the start of the echo by the change of color from blue to red and an enlargement of this zone is included. Thus, the threshold method is very simple but imprecise: this method is affected by the signal amplitude and when there is noise or perturbations the threshold must be increased and this produces a displacement of the start time considered.

**Figure 13.**
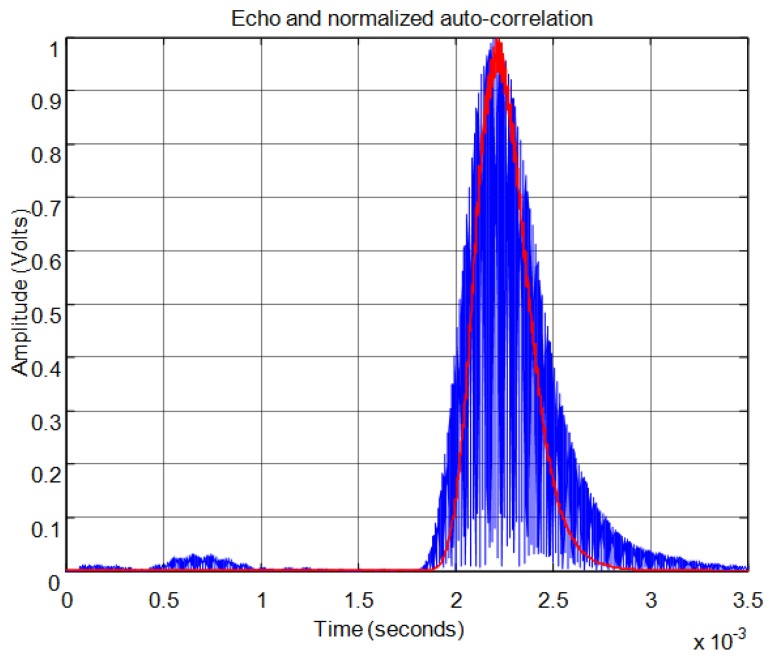
Normalized echo and maximum auto-correlation.

**Figure 14.**
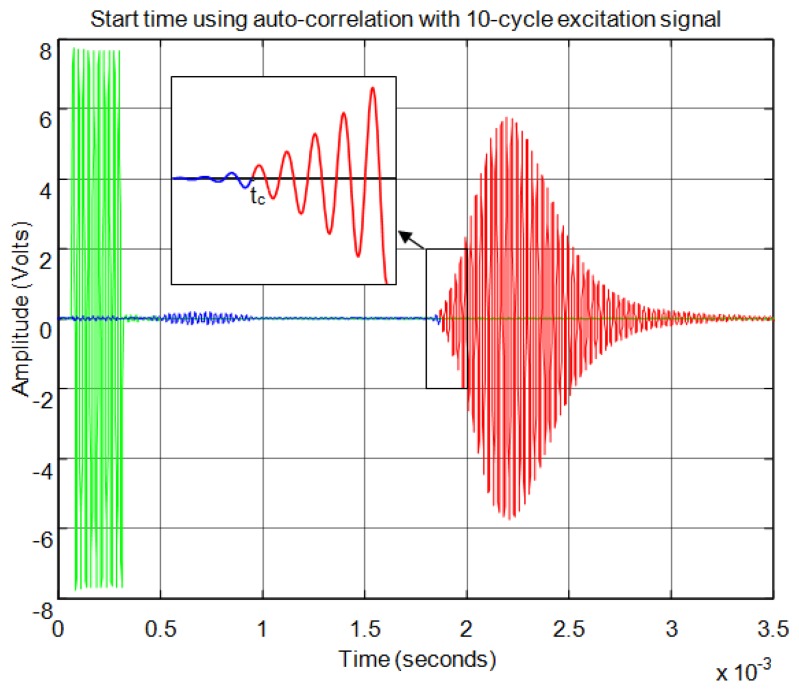
Start time of the echo using auto-correlation.

For the correlation method, in order to calculate the start time of the echo, the signal must overcome a minimum level of auto-correlation in the received echo. In Figure 13 the echo (in blue) and the maximum value of the auto-correlation (in red) can be seen, considering a time window of one cycle of signal for each sample. In Figure 13 the noise derived from the direct reception of the excitation signal located between 0.5 and 1 ms of the response can be seen. Here, it can be observed that no auto-correlation is produced. Therefore, the level of auto-correlation in order to detect the beginning of the echo can be very low. However it can be appreciated that it takes several cycles of the echo to detect a noticeable level of auto-correlation. With one percent of the maximum normalized auto-correlation level, Figure 14 shows, by the change in color from blue to red and an enlargement of this zone, the point detected as the beginning of the echo. It can be observed that the detection of the beginning of the echo is more accurate than with the threshold method.

For the proposed method, in order to calculate the start time *t_m_* of any echo, the time of maximum *t_max_* of the echo is used. First, once the number of cycles of the excitation signal and the time constant of the sensor employed are known, using expressions (15) and (16) corresponding to the envelope of the echo, the maximum value of the echo *y_max_* can be located, and the time in which this maximum is produced *t(y_max_)* can be obtained. Expression (15) covers the range of time from 0 to n·T seconds, *i.e.*, the duration of the excitation signal. As the maximum always occurs after the end of the excitation, for *t* > *n*·*T*, expression (16) can be directly used to find the maximum and its corresponding time *t(y_max_)*. This time will be constant for any echo while the sensor type and the number of cycles of the excitation do not change.

Thus, once the time elapsed between the maximum and the start of the echo is calculated, the start time *t_m_* can be calculate (see Figure 10) based on the maximum time *t_max_* using:

(18) $$t_{m}=t_{\text{max}}-t\left(y_{\text{max}}\right)$$

This method always takes into account the echo with the highest pressure of any echoes that the signal presents, discarding any noise which could be considered as the start of the real echo, e.g., the noise derived from the direct reception of the excitation signal already mentioned.

**Figure 15.**
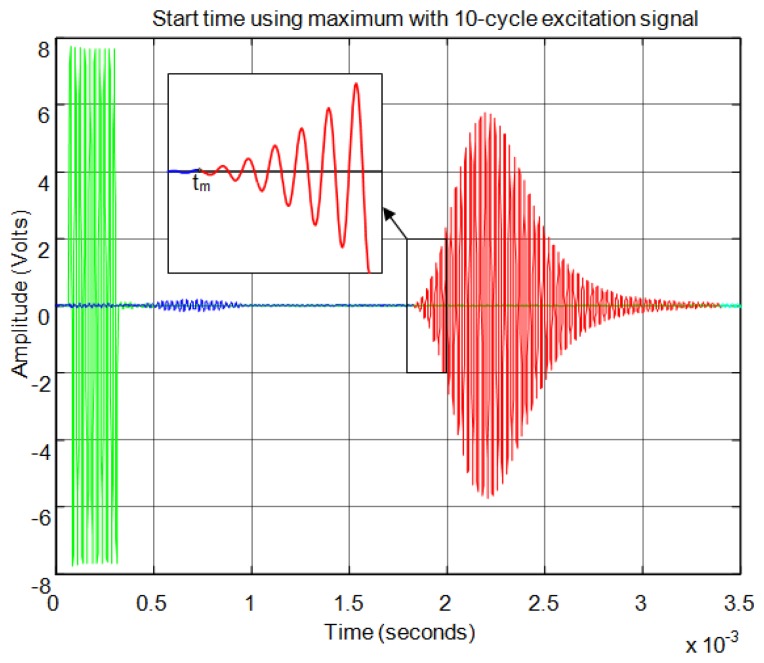
Start time of the echo using maximum.

**Figure 16.**
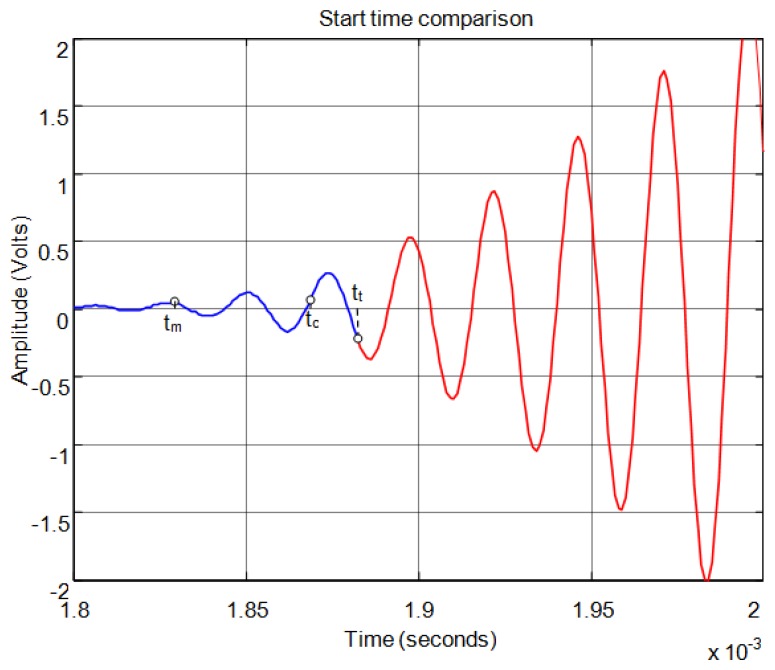
Comparison of the start time obtained by threshold t_t_, auto-correlation t_c_ and maximum t_m_.

Figure 15 shows the start time of the echo using the time of maximum signal. The start time obtained is much more accurate than is shown in Figures 12 and 14 for threshold and auto-correlation respectively. Figure 16 shows in detail the start points of the echo estimated by each method.

**Figure 17.**
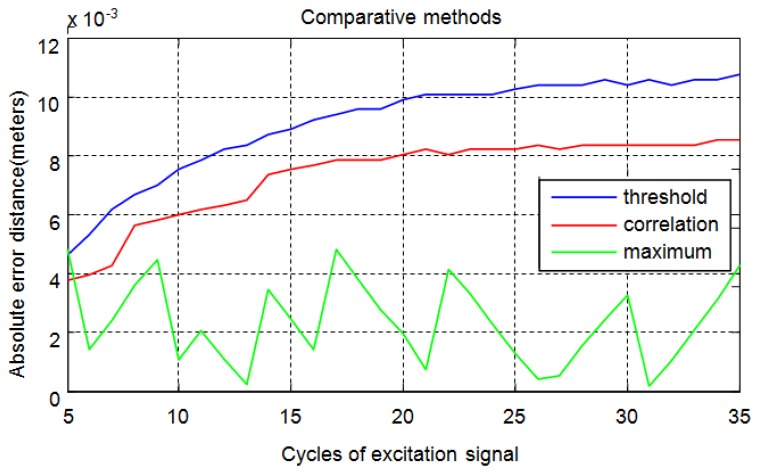
Comparison of absolute average errors in the distance calculation.

In summary, Figure 17 shows a comparison of absolute average errors obtained using the three methods to calculate distances from the time of flight for echoes obtained by an excitation signal that varies from five to thirty-five cycles.

**Figure 18.**
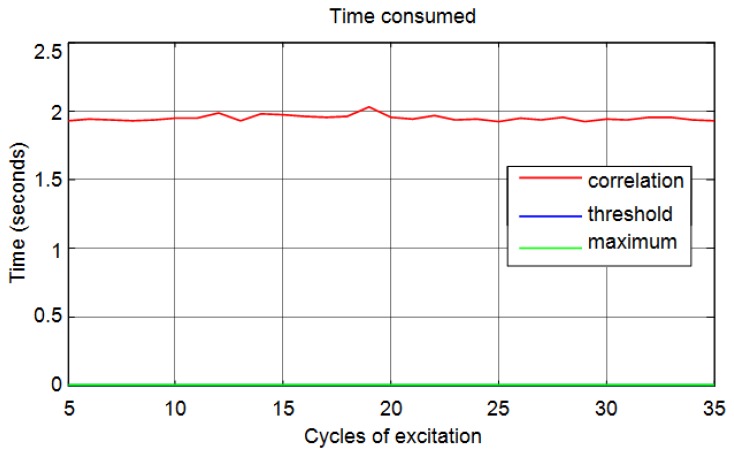
Computation time for the calculation of distances.

Another important aspect is the calculation time of the distances based on time of flight. Figure 18 shows a comparison of the time consumed for the three methods corresponding to the cases previously analyzed (Figure 17).

**Figure 19.**
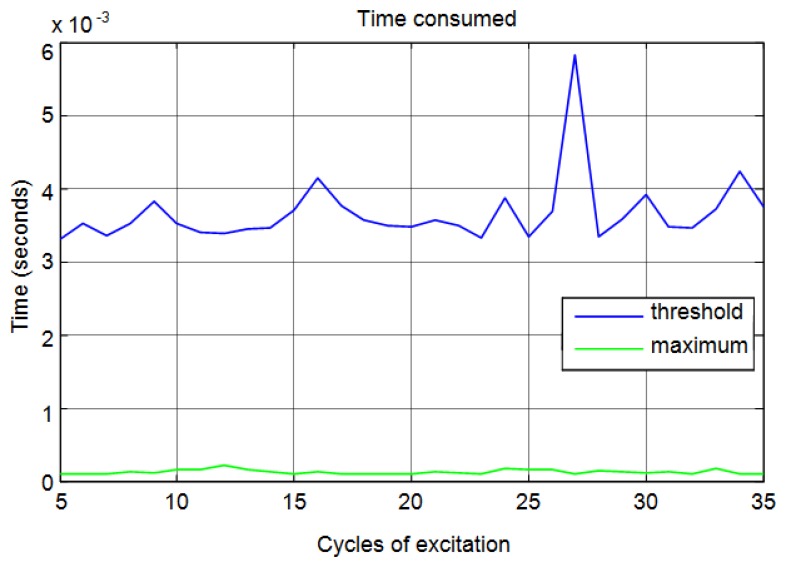
Computation time for the calculation of distances for threshold and maximum methods.

It can be seen than correlation is a time-consuming method compared with the threshold and maximum methods. In Figure 19, in order to obtain a better visualization, only the time taken for threshold and maximum methods are shown. You can see that the proposed method is less time-consuming. This reduction in time is because the method only needs to detect the maximum of the signal to calculate the start time.

### 4.2. Time of Flight with Multiple Overlapping Hidden Echoes

The model developed in the previous section can be used to obtain the time of flight in a fast and accurate way. This method enables accurate separation of signal and noise and so correctly identifies the main echo in the receptor avoiding obtaining false distances.

This method also allows a precise calculation of the start time of the echo when the complexity of the received signal is high, as in the case of hidden overlapping echoes.

This type of situations is normally found, for example, when a mobile robot approaches a corner (*i.e.*, the junction of two walls forming a concave surface). In this situation, the receiver sensor simultaneously captures the echoes from the rebounds off the two walls. Depending on the shape of the corner and the relative position of the sensors with respect to the corner, more echoes may appear simultaneously. This situation occurs when second rebounds exist. It may happen when the echo generated by the emitter, first bounces off one of the walls, then bounces off the other wall and is finally captured by the receiver sensor.

**Figure 20.**
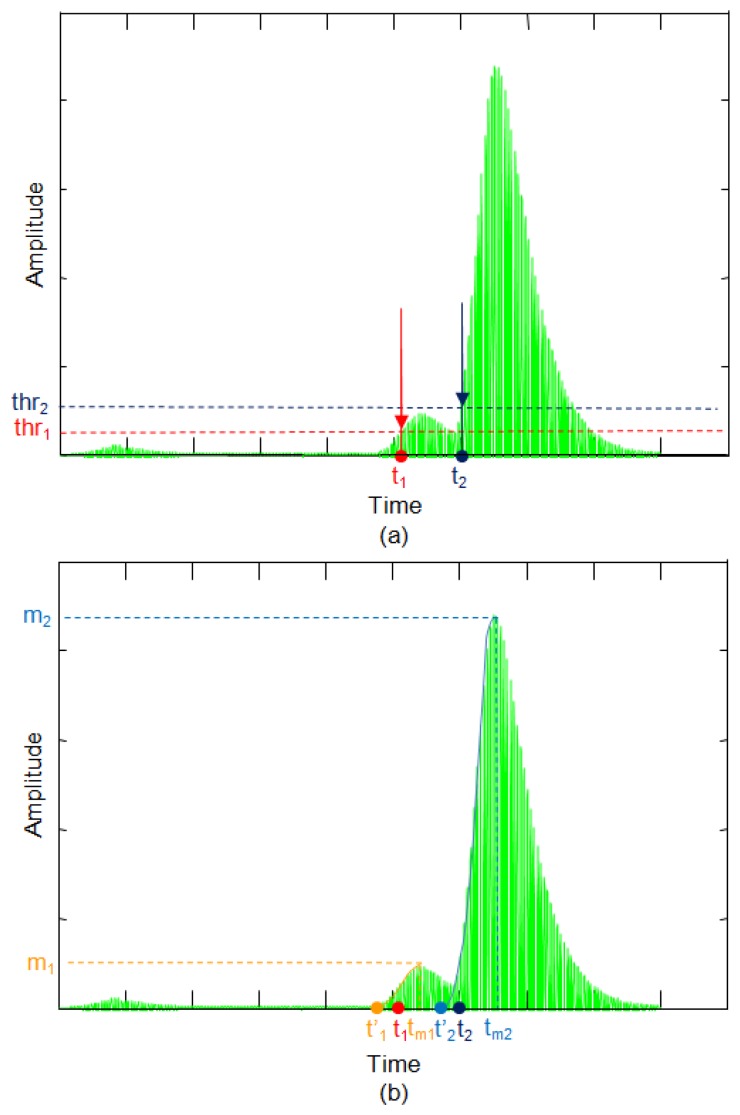
Comparison of methods: (**a**) threshold; (**b**) maximum.

In Figure 20, the threshold method is compared with the proposed method for an echo formed by two overlapping sub-echoes in the presence of noise. Figure 20a shows the start of the echo estimated with the threshold method. The start time t_1_ is obtained by selecting a threshold signal thr_1_ that exceeds the noise level. With this threshold value the starting time t_1_ is obtained. When you want to calculate the start time for the echo with the greatest amplitude, it would be necessary to set a threshold thr_2_, which exceeds the first echo level. With this method only one starting point is obtained whether the echo is formed by one or more sub-echoes.

The threshold method always finds the first echo which exceeds the threshold, regardless of which echo has the greatest or least amplitude. This condition translated to distance means that this method always finds the nearest object regardless of whether the object is the main object in the scene or not.

In Figure 20b the start time is calculated based on maximum value detection. Therefore, first, the calculus is not affected by the noise level and, second, this method allows simultaneous detection of the starting point of several overlapping echoes. The number of echoes is set by the number of maximum analyzed. So in this example, considering two sub-echoes, two relative maxima, m_1_ and m_2_, and the corresponding times t_m1_ and t_m2_ are detected. From these maximum times the starting points of the two echoes, t'_1_ and t'_2_ are estimated. As shown in Figure 20b, these times are more accurate than the times t_1_ and t_2_ calculated by the threshold method.

Therefore, in summary, the proposed method, besides allowing better precision, also provides further information since it detects whether there are multiple overlapping echoes. The existence and detection of multiple simultaneous echoes carries intrinsic information that several objects are being detected simultaneously. This information is highly valuable for example when it is used in autonomous navigation of robots, as it gives much more complete information about the robot's environment, allowing several distances to the closest points to the robot to be obtained simultaneously.

**Figure 21.**
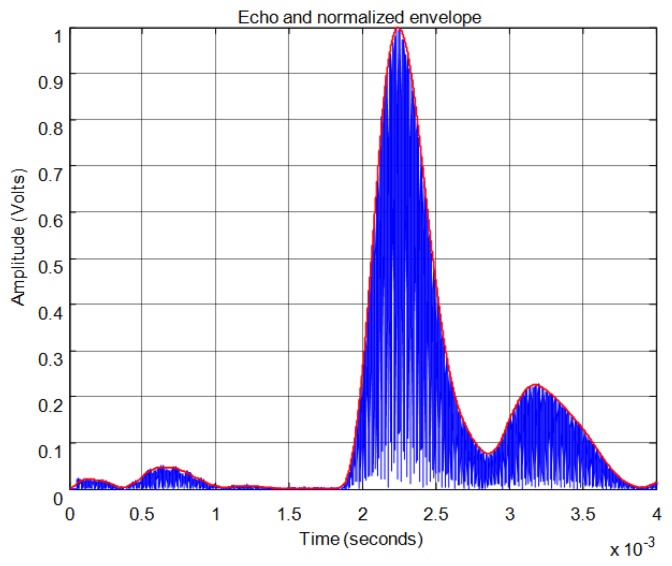
Normalized echo and envelope for an echo formed by two overlapping sub-echoes.

**Figure 22.**
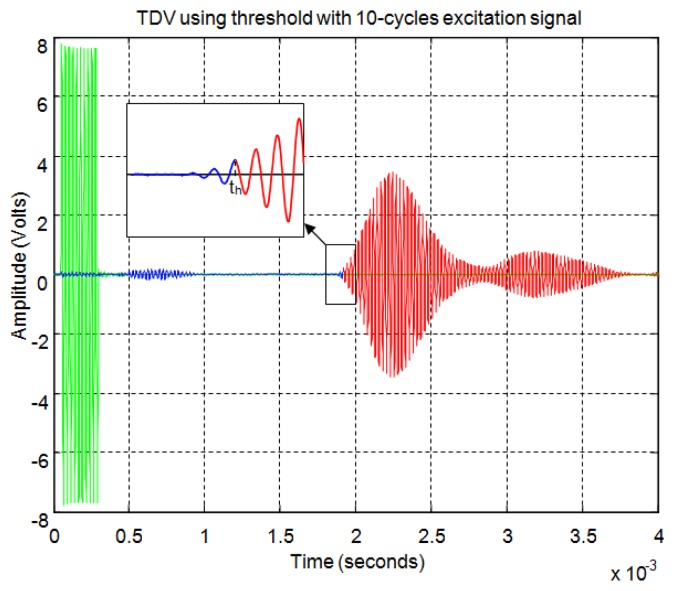
Start time using threshold for an echo formed by two overlapping sub-echoes.

**Figure 23.**
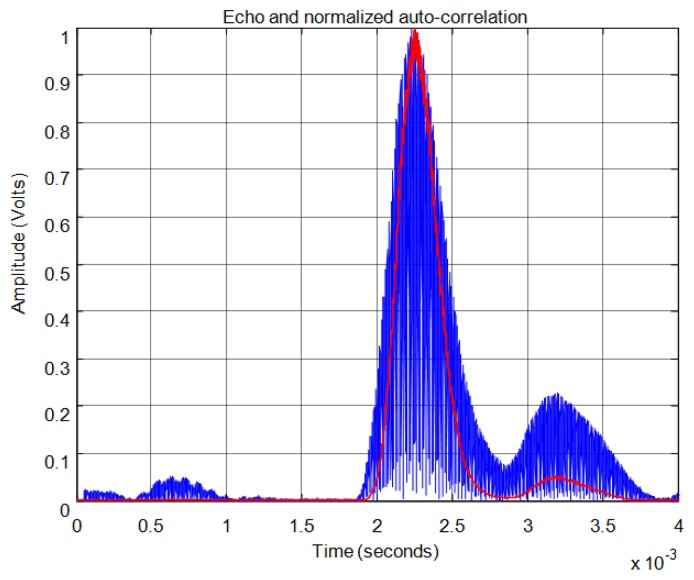
Normalized echo and auto-correlation for an echo formed by two overlapping sub-echoes.

**Figure 24.**
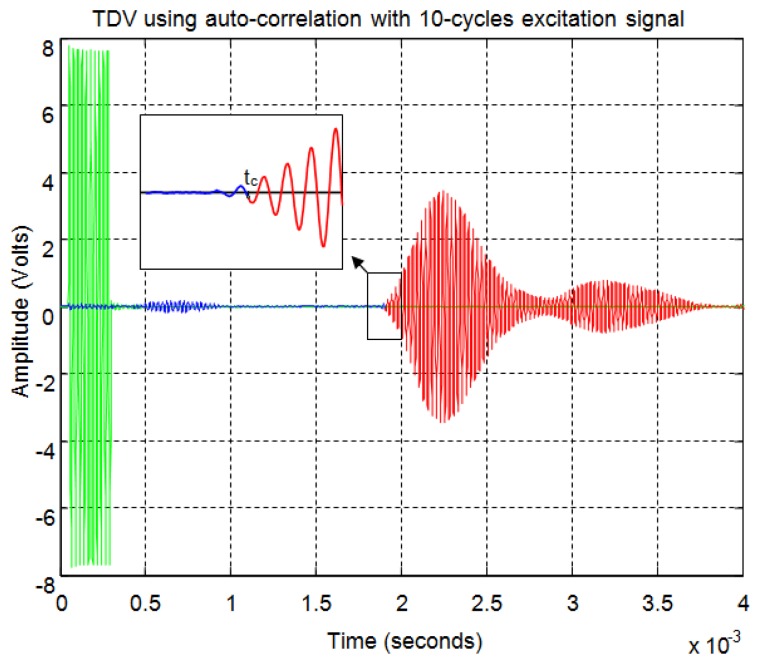
Start time using auto-correlation for an echo formed by two overlapping sub-echoes.

**Figure 25.**
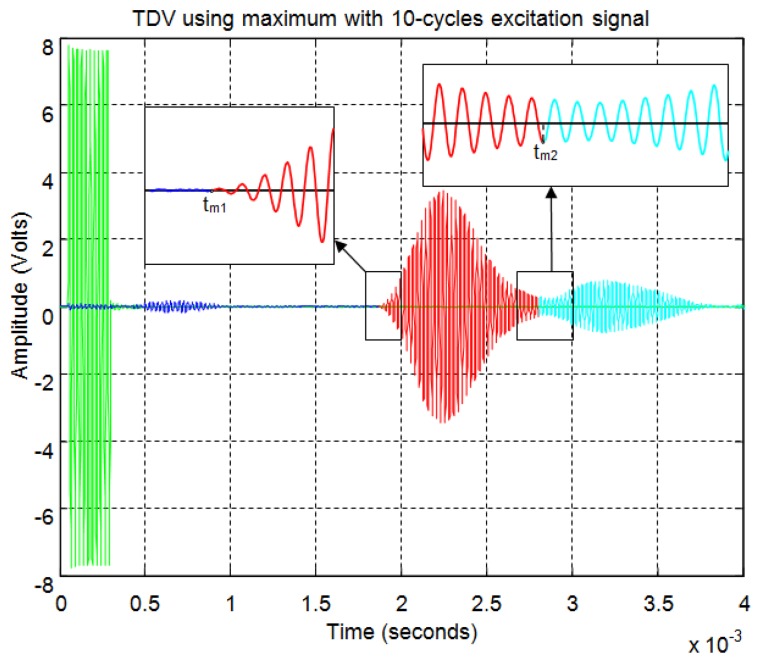
Start time using maximum for an echo formed by two overlapping sub-echoes.

Figure 21 shows an example for an echo formed by two overlapping sub-echoes. The figure shows the envelope employed for obtaining the start time using a threshold which exceeds the noise level. With this threshold, Figure 22 shows the beginning of the echo at the point of change from blue to red. Figures 23 and 24 show the same case using the auto-correlation method. Figure 25 shows the two starting points, change from blue to red and change from red to cyan, corresponding to the two overlapping echoes using the maximum method. Figure 25 shows that starting point of each echo differs from the point where there is the minimum signal level.

**Figure 26.**
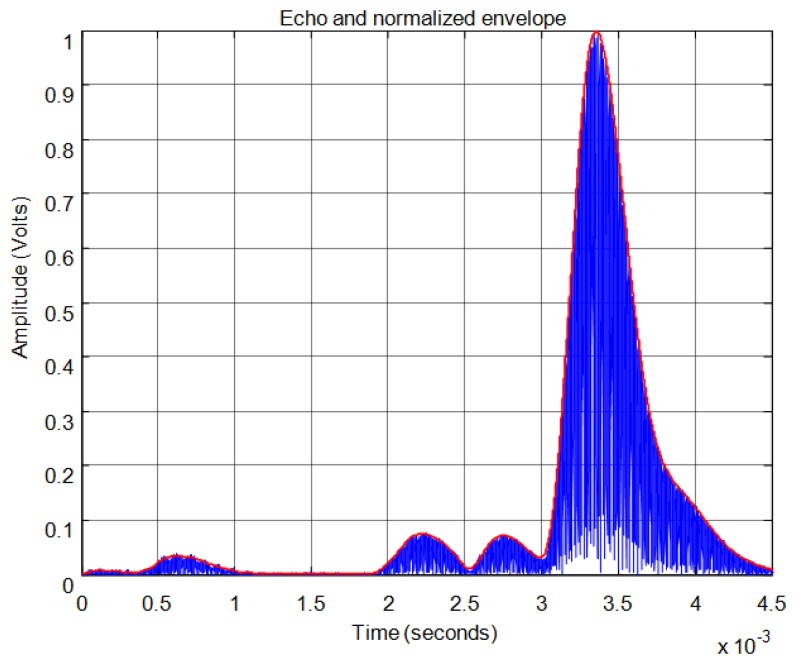
Normalized echo and envelope for an echo formed by three overlapping sub-echoes.

**Figure 27.**
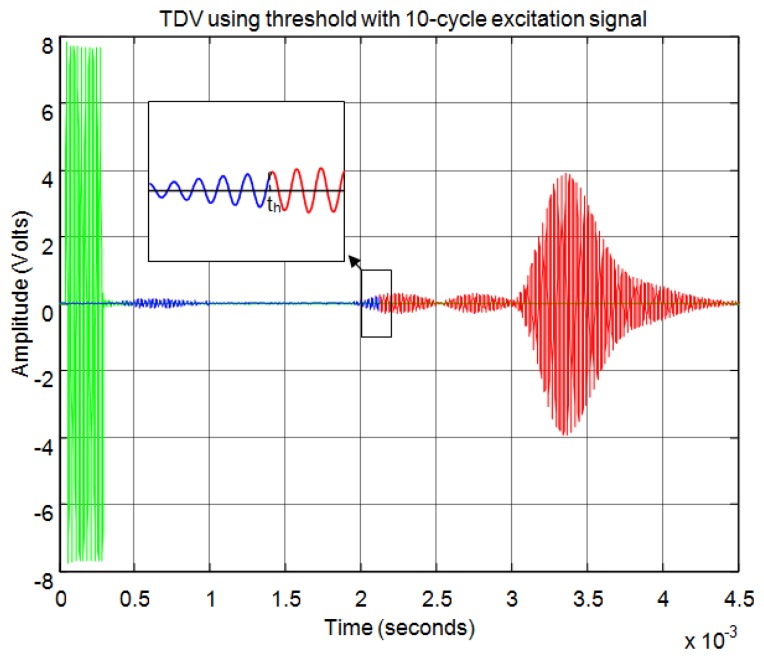
Start time using threshold for an echo formed by three overlapping sub-echoes.

**Figure 28.**
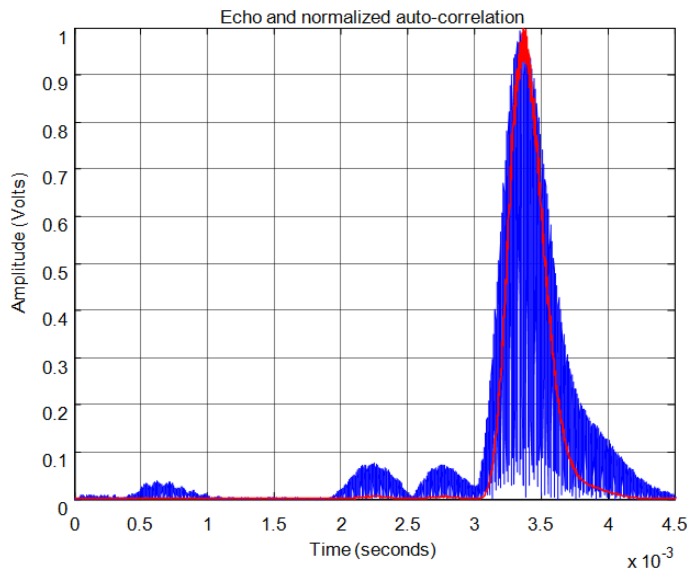
Normalized echo and auto-correlation for an echo formed by three overlapping sub-echoes.

**Figure 29.**
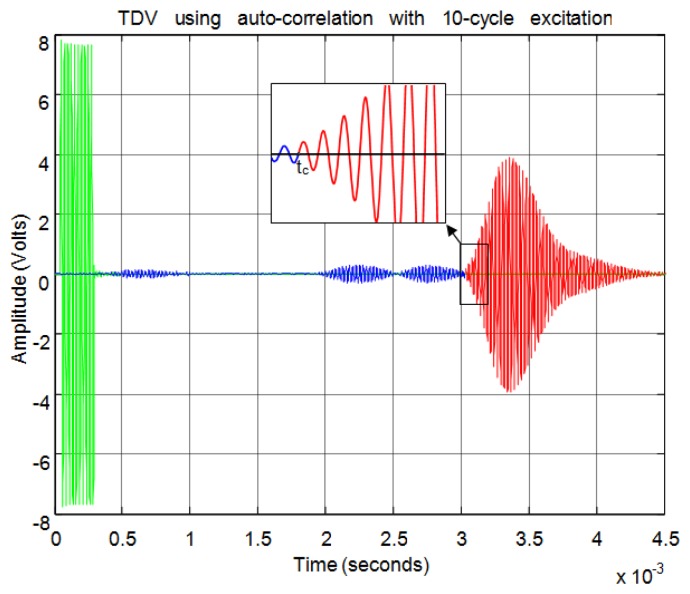
Start time using auto-correlation for an echo formed by three overlapping sub-echoes.

**Figure 30.**
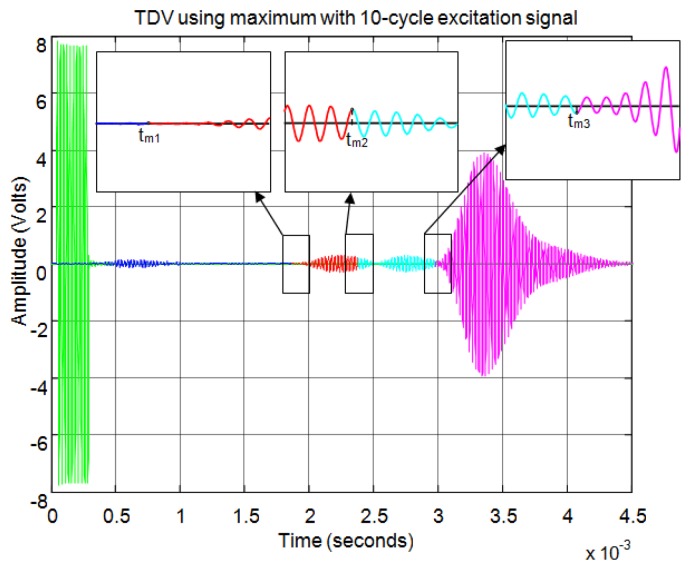
Start time using maximum for an echo formed by three overlapping sub-echoes.

**Figure 31.**
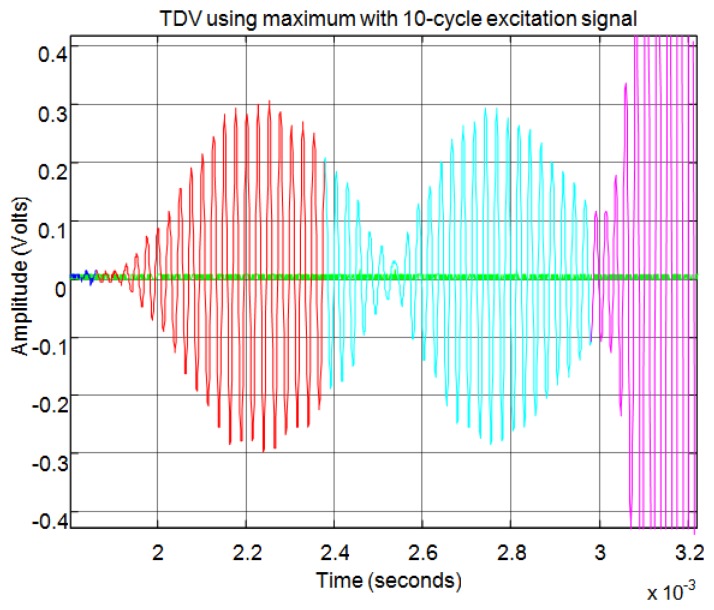
Detail of the start time of the three overlapping sub-echoes.

Figures 26, 27, 28, 29, 30 and 31 show another example, in this case formed by three overlapping sub-echoes. In this case Figure 27 shows that the threshold method detects the first sub-echo because this exceeds the established noise level. This sub-echo corresponds to a low signal echo. In contrast, Figure 28 shows that the level of auto-correlation for the first two sub-echoes is very low. Therefore, this method detects only the largest sub-echo as shown in Figure 29.

Figure 30 shows how the maximum method correctly identifies the starting point of the three sub-echoes. Once again, the starting point does not coincide with the point of minimum level of signal when several sub-echoes are overlapping. With the maximum method the signal level established to distinguish between noise and echo does not affect the starting point of the echo, as can be seen comparing Figures 27 and 29 with Figure 30.

Figure 31 shows an enlargement of the zone of the start of the three sub-echoes. You can observe that the starting point is not affected by the level of the echo signal, having the same accuracy for low signal echoes and for high signal echoes. Finally, this method enables the exact start time of each sub-echo to be set although these appear partially overlapping. This effect can be clearly seen in Figure 31, where the second echo (in cyan) begins before the first echo (in red) extinguishes, so that the starting point does not corresponds to the minimum signal level between the two echoes.

## 5. Conclusions

A study of reflection and propagation of ultrasonic waves in air generated by low-frequency sensors with application to the autonomous navigation of mobile robots in structured environments has been performed.

Based on this study, a model of the transient response has been proposed in order to estimate the signal captured by the receiver for the cases of direct rebound on a flat wall and composite rebounds from any type of corner, including those that are not right angles. This model has been defined based on the general solution of the wave equation, which has been particularized for the characteristics of the propagation medium and of the sensor, namely, the radiation pattern and the dynamics of the response-to-pulse type excitation signals.

This model enables the response of the ultrasonic sensors to be found for use in applications like mobile robotic and allows the inverse process to be subsequently applied for locating the relative position of the walls and corners from the information of the sensors.

Moreover, this model permits the definition of a new method to estimate the start time of the echo in order to calculate the time of flight. This method, based on the maximum time, is not affected by the amplitude of the signal, is more robust to noise and interferences and also enables the method to work with overlapping of echoes, finding a start time for each peak that exists in the signal.

In summary, this new method permits several start times to be obtained simultaneously when the received signal is formed by several overlapping echoes. The classical methods only obtain the first time of flight, whether the first echo is the most important or not. Thus, the proposed system to calculate the start time permits the extraction of more information based on one single received signal. For example, for autonomous navigation, this method enables several rebounds from several objects to be registered simultaneously.
